# Patient Selection Criteria and Procedural Standardization for Carotid Artery Stenting—A Single Center Experience

**DOI:** 10.3390/jcm12103534

**Published:** 2023-05-18

**Authors:** Paolo Calo, Alexander Oberhuber, Hartmut Görtz

**Affiliations:** 1 Department of Vascular Surgery, Bonifatius Hospital Lingen, 49808 Lingen, Germany; 2 Department of Vascular and Endovascular Surgery, University Hospital Muenster, 48149 Muenster, Germany

**Keywords:** CAS, complication, anatomy, standardization, outcome, stroke

## Abstract

The gold standard for the treatment of carotid artery stenosis is the carotid endarterectomy (CEA). According to current guidelines, carotid artery stenting (CAS) is an alternative. Randomized control trials (RCTs) show significantly higher rates of peri-interventional strokes after CAS compared to CEA. However, these trials were usually characterized by a great heterogeneity in the CAS procedure. In this retrospective analysis from 2012 to 2020, 202 symptomatic and asymptomatic patients were treated with CAS. Patients were carefully pre-selected according to anatomical and clinical criteria. In all cases, the same steps and material were used. All interventions were performed by five experienced vascular surgeons. Primary endpoints of this study were perioperative death and stroke. Asymptomatic carotid stenosis was present in 77% of the patients and symptomatic in 23%. The mean age was 66 years. The average degree of stenosis was 81%. The CAS technical success rate was 100%. Periprocedural complications occurred in 1.5% of cases, including one major stroke (0.5%) and two minor strokes (1%). The results of this study indicate that through a strict patient selection based on anatomical and clinical criteria, CAS can be performed with very low complication rates. Furthermore, standardization of the materials and the procedure itself is crucial.

## 1. Introduction

Stroke is the third leading cause of death in Germany [1]. More than 80% of strokes are due to ischemic causes [2]. In about 15% of ischemic strokes, stenosis or occlusion of the carotid artery is the cause. This represents about 30,000 new carotid-associated strokes per year in Germany [3].

The gold standard for the treatment of stenosis of the internal carotid artery is the carotid endarterectomy (CEA). However, according to the current version of the AHA (i.e., European and German guidelines), carotid stenting is an alternative to CEA [3,4,5]. Nevertheless, in several randomized control trials (RCTs), CAS showed significantly higher rates of peri-interventional stroke compared to CEA [6,7,8,9]. In contrast, many retrospective single-center studies showed significantly better outcomes after stenting [10,11,12]. The cause of this discrepancy is not entirely clear. Presumably, the difference is due to the nature of the studies. In randomized trials, patients with any anatomy type were included, and in the multi-center trials there were significant differences in the interventions in terms of the type of material which was used, the procedure itself, and the experience of the interventionalist.

The primary aim of this study was to check whether a critical selection of patients, according to anatomical and clinical criteria combined with a standardized treatment protocol, could lead to safe and effective stenting of the carotid artery.

## 2. Materials and Methods

### 2.1. Study Design

This study is a retrospective, non-randomized, single center study.

### 2.2. Patient Selection, Inclusion and Exclusion Criteria

For this purpose, we examined all of our patients who underwent a transfemoral stenting of the internal carotid artery at our institution from 2012 to 2020. Patients with restenosis, radiation-induced stenosis, aneurysms, isolated common carotid artery stenosis, and tandem stenosis proximal to the carotid bifurcation were excluded. During the nine years under investigation, a total of 719 patients underwent carotid repair for both symptomatic and asymptomatic carotid lesions. From the total number of patients, 497 received CEA and the remaining 222 received CAS. Based on the criteria mentioned above, some patients were excluded from this study.

A total of 202 symptomatic and asymptomatic patients was included in the study. The degree of stenosis was determined according to the NASCET criteria. Inclusion criteria were: for symptomatic patients, ≥50% degree of stenosis of the internal carotid artery (ICA); and for asymptomatic patients, ≥70% stenosis. The degree of stenosis was determined by a consultant neurologist or vascular surgeon using duplex ultrasound (DUS). DUS was supplemented by computerized tomography angiography (CTA) or magnetic resonance angiography (MRA) to evaluate the aortic arch (AA) and the extra- and intracranial vessels.

In our institution, the endovascular approach is considered first-line treatment in all patients with appropriate clinical and anatomical criteria. Anatomic inclusion criteria are: an AA type I-II, an angle between the aortic arch (AA) and the supra-aortic vessel between 45–110°, an angle between the common carotid artery (CCA) and ICA of <60°, and the absence of course abnormalities such as kinking or coiling. If any of these criteria were not met, CEA was carried out.

### 2.3. Endpoints

Primary endpoints were the combination of death and stroke during the in-hospital stay. Secondary endpoints were myocardial infarction, access-related complications, and nerve lesions.

### 2.4. Analyzed Criteria

The anatomical and clinical criteria that have been analyzed in this study come from an exhaustive investigation of the existing literature.

#### 2.4.1. Anatomical Criteria

**Figure 1:**
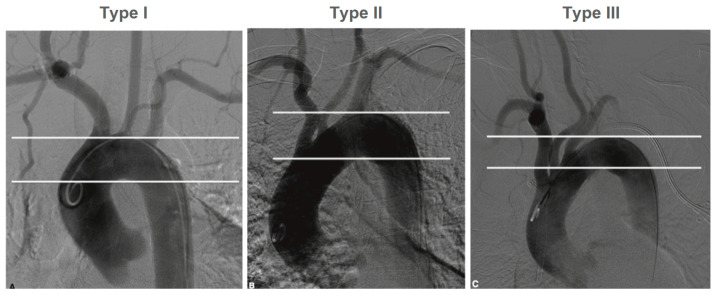
Type I, II and III aortic arch.

**Figure 2:**
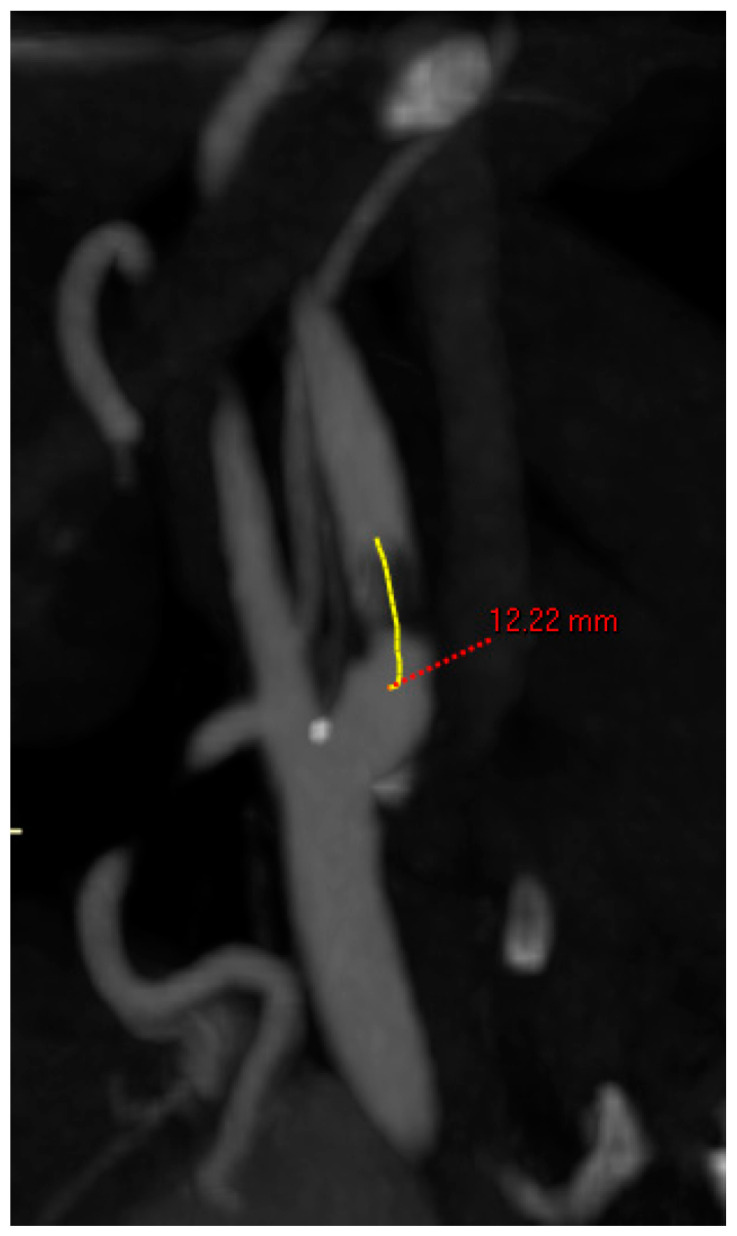
Centerline measurement of the length of the lesion.

**Figure 3:**
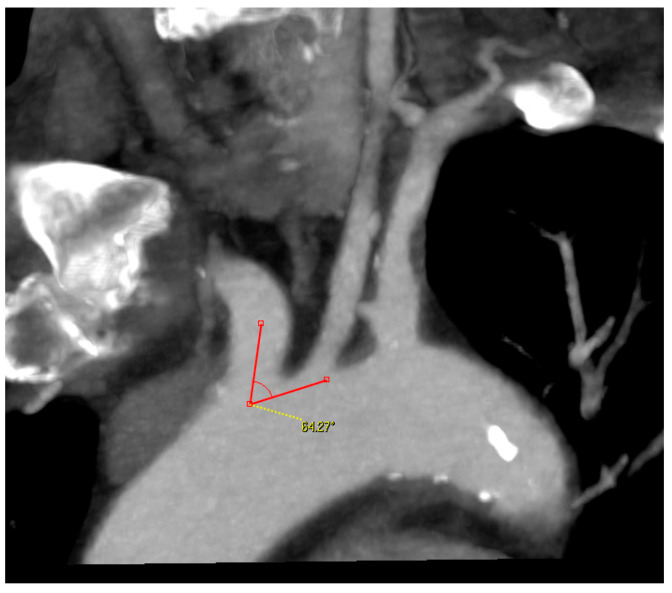
Angle measurement between the aortic arch and supra-aortic vessel.

**Figure 4:**
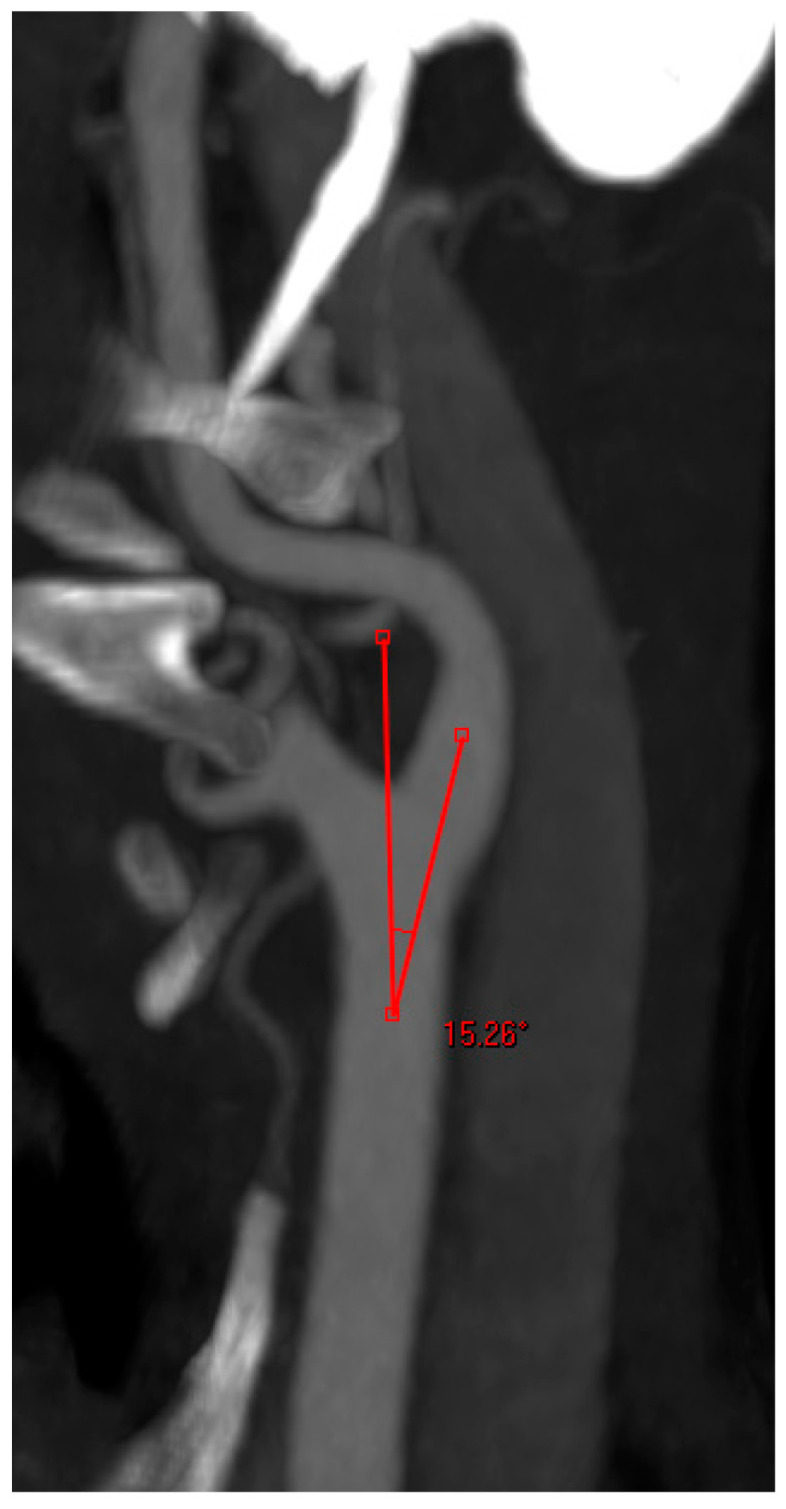
Angle measurement at the carotid bifurcation.

**Figure 5:**
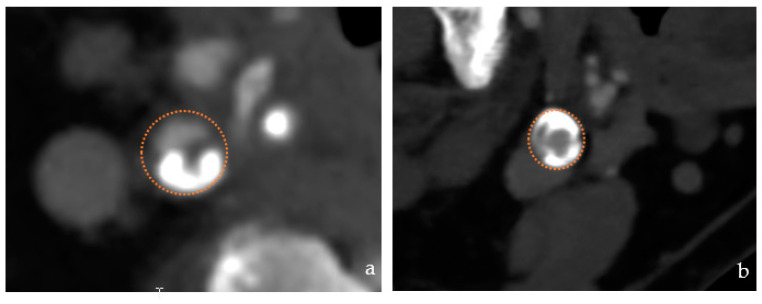
Lesion type (**a**) Eccentric lesion (**b**) concentric lesion.

The following anatomic criteria were evaluated:

- **Degree of stenosis:** according to the NASCET criteria;
- **Aortic arch type:** type I and II (Figure 1);

- **Side of lesion:** right or left;
- **Length of lesion:** centerline measurement in mm of the entire length of the atherosclerotic lesion (Figure 2);

- **Diameter of the CCA and ICA:** in mm;
- **Angle between the AA and the supra-aortic vessel:** measured on the treated side only, through a multiplanar reconstruction and visualization of the AA in coronary and left anterior oblique (LAO) projection. One arm of the angle was drawn along the outer curvature of the AA and the second arm was placed in the middle of the CCA or brachiocephalic trunk. The right-side angle was chosen for selection criteria (Figure 3);

- **Angle between the CCA and the ICA:** measured on the treated side only. At the carotid bifurcation, one arm of the angle was placed along the central axis of the CCA and the other arm was drawn into the central axis of the ACI. The cranial angle was measured (Figure 4);

- **Elongation, kinking and coiling:** Elongation was defined as a curvature of the vessel >90° and kinking <90°. Coiling formation is defined as loop;
- **Plaque morphology:** if a CT scan was performed preoperatively, the plaque morphology was recorded by measuring Hounsfield units (HU). The vast majority of our patients received a CTA as secondary image modality. For the patients on whom an MRA was performed, plaque evaluation according to the HU was not possible. According to the HU, the plaques were classified as lipid core (−100–49 HU), fibrous (50–149 HU), or calcified (150–1300 HU) [13];
- **Lesion type:** target lesions were categorized as eccentric, concentric, or carotid near occlusion (>90° stenosis) (Figure 5).

#### 2.4.2. Clinical Criteria

In addition, the following clinical data were collected:

- **Cardiovascular risk profile:** arterial hypertension, diabetes mellitus, hyperlipidemia, smoking, and cardiac history;
- **Classification according to symptoms:** asymptomatic or symptomatic if neurological-associated symptoms occurred in the previous 6 months;
- **Gender:** male or female;
- **Age;**
- **Preoperative medication:** antihypertensive therapy, antiplatelet therapy and statins.

All 202 patients were treated according to strict surgical standards with homogeneous materials. Only the Carotid Wall stent together with the Filter Wire EZ (Boston Scientific Corp., Marlborough, MA, USA) were used. Postoperatively, all patients received a dual antiplatelet therapy, combining ASS with clopidogrel for 8 weeks. Furthermore, all interventions were exclusively performed by only five experienced vascular surgeons having over 10 years of experience in the field of central and peripheral endovascular procedures.

All patients were examined pre- and post-operatively by a consultant neurologist. Confirmed strokes were subdivided according to clinical presentation, location, and modified Rankin Scale [mRS]. Minor stroke (mRS 0–2) refers to non-disabling strokes, and major strokes (mRS 3–5) refers to disabling strokes.

### 2.5. Statistical Analysis

Continuous variables are expressed as mean ± standard deviation for parametric data and median with interquartile range for non-parametric data, whereas dichotomous variables are presented as crude numbers and percentages. Normally-distributed data were compared using the Student’s *t* test, and non-normally distributed data with the Mann-Whitney U test. Categorical data were analyzed using the chi-square and Fisher’s exact test. Univariate or multivariate analyses were used to examine the influence of individual factors. The significance level was assumed to be *p* < 0.05. All statistical analyses were performed using SPSS Statistics for Windows version 26.0 (IBM Corp., Armonk, NY, USA).

## 3. Results

A total of 202 CAS interventions were performed. The majority of patients were male, and more than two-thirds were asymptomatic. The mean age was 66 years (min 41—max 95). The cardiovascular risk factors and the medication of our patients are shown in Table 1. Ninety-two (46%) of our patients had a history of coronary-artery disease. From this group, 59 (30%) patients had already been through a percutaneous coronary intervention and 25 (12%) through a coronary artery bypass graft operation before CAS. Only for 8 (4%) of these patients was the diagnosis of coronary artery disease established through noninvasive procedures.

The anatomic features are summarized in Table 2. Right-sided lesions were 20% more frequently intervened. The mean degree of stenosis was 81 ± 11%. The mean stenosis length was 25 mm. The mean diameter of the CCA was 7 mm and of the ICA was 5 mm.

Type II AA was found 20% more frequently than type I AA. The angle between the AA and the supra-aortic vessel varied depending on the type of AA and the side of the lesion. Type I AA and right-sided lesions were associated with larger angles, whereas type II AA and left-sided lesions were associated with smaller angles.

All patients underwent DUS, and a second imaging modality CTA was performed in 71% of cases and an MRA in 29%. According to our plaque-morphology assessment, the majority of patients showed plaque lesions that were composed of either a lipid core or fibrous tissue. Two thirds of complications occurred in plaques consisting of a lipid core.

Regarding the low number of enrolled patients in this study and the very low perioperative complication rate, the results of the different statistical methods applied to the whole cohort revealed no significant predictor for the primary endpoints. The *p* value was on every analyzed criteria *p* > 0.05.

**Figure 6:**
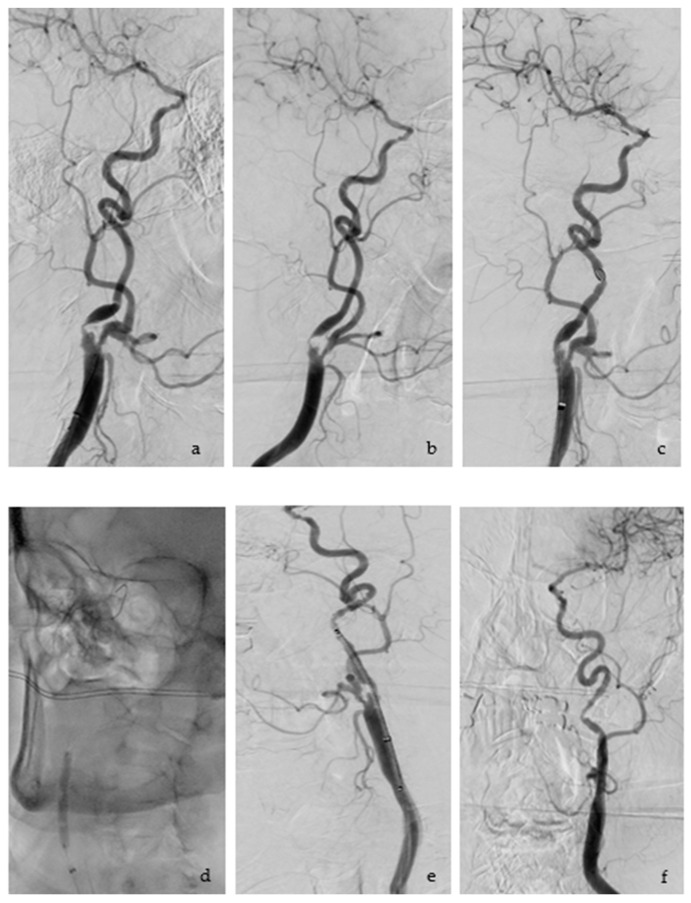
CAS Procedure: (**a**) showing the anterior-posterior view of the stenosis of the ICA; (**b**) lateral projection of the stenosis; (**c**) the stenosis has been crossed with the filter system, this can be recognized as a round radiopaque object above the stenosis; (**d**) carotid angioplasty; (**e**) post angioplasty, stent positioning and measurement before final implantation; (**f**) angiography control after stent has been deployed.

The technical success rate for this study was 100%. Figure 6 shows the steps of the CAS Procedure.

During the hospital stay, three patients developed acute neurological symptoms. One of them suffered a major stroke and the two others minor strokes. All complications occurred in the asymptomatic group, resulting in a complication rate of 1.9% in this cohort. No neurologic complications occurred in the symptomatic group, so the overall perioperative complication rate for this study was 1.5%.

One patient suffered an ipsilateral retinal ischemia, which was categorized as a major stroke (mRS3). This occurred during CAS intervention. Subsequently, the patient was transferred to an ophthalmologic maximum-care center. Despite all therapy attempts, vision could not be restored. This patient was under 70 years of age, and due to existing medical conditions, was considered as an ASA-3 (American Society of Anesthesiologists, Schaumburg, IL, USA) patient. A history of 3-time coronary artery bypass surgery and chronic obstructive pulmonary disease was present. Additionally, there was a 90% contralateral carotid artery stenosis.

The second patient, who was designated as suffering a minor stroke, suffered an amaurosis fugax due to occlusion of the internal carotid artery at the level of its petrous course via an intraoperative embolism. This was resolved through a bail-out maneuver which consisted of an aspiration thrombectomy followed by local i.a. thrombolysis (5 mg bolus rTPA, followed by continuous lysis therapy for another 2 h). Postoperatively, no persistent neurological symptoms could be detected in this patient, so further imaging was omitted. This patient was over 70 years old, and besides an untreated Hypertriglyceridemia there were no other comorbidities or risk factors present.

Another patient complained of paresthesia in the right upper extremity and right side of the face. DWI-MRI revealed multiple small embolic infarctions in the left hemisphere, in the internal capsule, and in the thalamus. The symptoms disappeared completely after a few days. This patient was over 70 years old and cardiovascular risk factors were absent.

The neurological events occurred despite the use of an embolic protection system (EPS). In this study, there were no periinterventional deaths, myocardial infarctions, or cranial nerve lesions.

Access-related problems such as pseudoaneurysms occurred only once in this study. This was surgically repaired by open repair.

**Figure 7:**
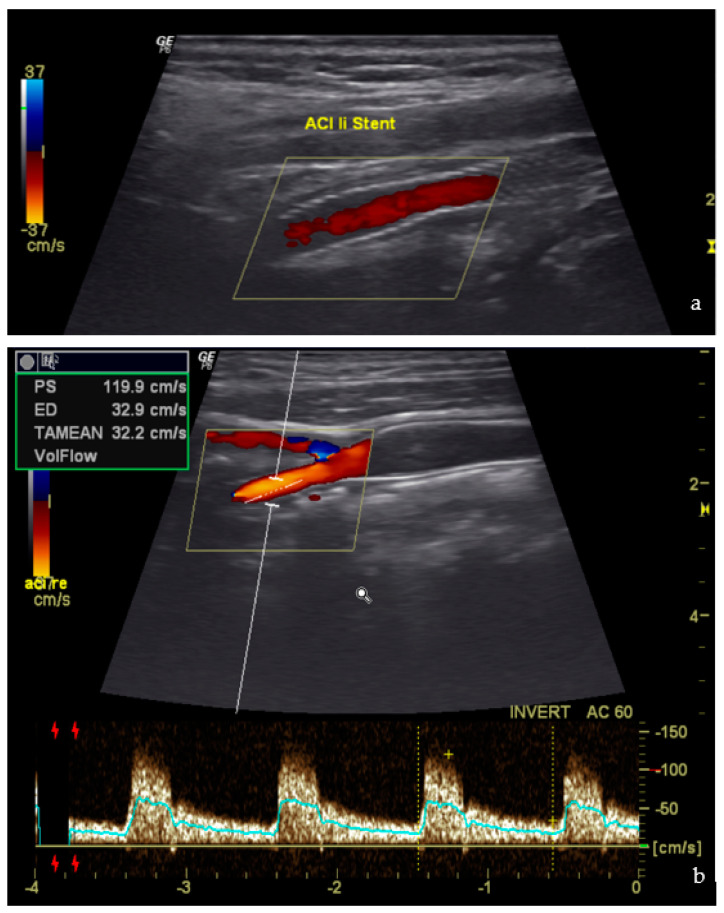
Postoperative ultrasound (**a**) Duplex of the Stent course (**b**) Doppler of the ICA.

Postoperative duplex ultrasound was performed on all patients before discharge. No signs of restenosis, carotid dissection, or thrombotic stent occlusion were evidenced. Figure 7 shows an example of normal findings on the postoperative duplex ultrasound.

Table 3 shows where the results of this study stand in comparison to the 30-day outcomes of randomized multi-center trials and single-center trials without randomization.

## 4. Discussion

The vast majority of international trials were conducted between 1990 and 2010. The first multi-center and randomized trials (CAVATAS 6.4%, SAPPHIRE 4.8%, EVA3-S 9.6%, ICSS 8.5%, SPACE 6.8%) showed initially unsatisfactory results in the periprocedural period [7,8,9,14,15]. Over time, CAS techniques and indications have evolved. Thus, at the end of this period, a lower perioperative complication rate was observed (CREST 5.2%, ACT-I 2.9%) [6,17].

Some studies distinguish between minor and major strokes using perioperative neurological complications [16,17]. Based on this distinction, comparable outcomes between CAS and CEA, in terms of disability or residual damage, could be achieved. As known, CAS has the disadvantage of causing more perioperative minor strokes. In contrast, CEA is more frequently associated with perioperative complications such as myocardial infarction, cranial nerve lesion, and hematoma. Bonati et al. showed, in a follow-up study of the ICSS trial, higher perioperative minor stroke rates after CAS but similar results at a follow-up average of 4.2 years, with 6.4% for the CAS and 6.5% for the CEA group. This demonstrated that CAS was primarily associated with a higher perioperative neurological complication rate, while during follow-up these complications were equalized between the two procedures [18]. These results were confirmed in a recently published study for asymptomatic patients through a retrospective sub-analysis of the CREST and ACT I trials. Even after an average of four years of follow-up, the complication rate for the combined endpoints of death, stroke, and myocardial infarction was 5.3% for CAS compared to 5.1% for CEA [19].

The perioperative incidence of stroke and death was lower in this study than in the RCTs that compared CAS against CEA for both symptomatic and asymptomatic patients. Based on our results, anatomical criteria should be taken into account when determining the indication for CAS. For cannulating the CCA, the angle between the AA and the supra-aortic vessel, as well as the AA-type, play very important roles. The AA changes over time, and these changes are caused, among other things, by atherosclerosis, arterial hypertension, and age. AA remodeling results in elongation of the AA and caudal positioning of the supra-aortic vessels [20]. At the same time, the difficulty of cannulating the supra-aortic vessels increases parallel to the AA-type, and simultaneously with an increased risk of embolization due to the required manipulations in the AA [21,22]. In our institution, a type III AA is considered a contraindication for transfemoral cannulation of the carotid artery.

The remodeling changes in the AA cause a variation in the angle between the AA and supraaortic vessels. Based on the available literature on this topic, it is still currently not possible to recommend an exact angle for CAS. Suh et al. reported that the angle between the AA and CCA is significantly smaller on the left side than with the brachiocephalic trunk. The left CCA originates from the AA and has an oblique course from right to left and from caudal to cranial. On the other hand, the CCA to the right side has a much straighter course [23]. We have generally treated moderate-grade angles, with no obtuse or acute angles. It was particularly interesting in this study that the angle on the left side was 10° smaller than on the right side. This confirms the assumption of Suh, G.Y. and is a possible explanation for why left-sided supra-aortic interventions are technically more difficult and are associated with higher complication rates [24,25]. Our results also showed that the angle of the type I AAs were greater than the type II AAs.

At the level of the carotid bifurcation, the angle between the CCA and ICA also plays an important role. Various studies support the statement that an obtuse angle at the carotid bifurcation can lead to a more difficult and risky cannulation of the ICA [25,26]. A subanalysis of the EVA-3S trial demonstrated that CAS interventions in patients with an angle >60° at the carotid bifurcation were associated with significantly higher complication rates [26]. An angle >60° was an exclusion criterion in this study. Our mean angle at the carotid bifurcation laid much lower than this value, and according to our experience an angle >60° at this level is relatively rare to find.

Regarding the stenosis, not only is the degree of luminal narrowing important, but so is plaque morphology and consistency. Two thirds of our perioperative neurological events had lipid-core lesions. In the third case, it was not possible to determine the plaque morphology because an MRA was performed as preoperative imaging. Our results tend to agree with those in the literature, where lipid-core lesions showed association with an increased risk of developing stroke [27].

With every 10% increase in the degree of stenosis, the risk of an ischemic stroke increases by approximately 26% [28]. Nowadays, CAS therapy is not recommended for the most severe stenosis or near occlusions [3,5]. These lesions are more difficult to recanalize and may cause inadequate stent deployment as well as possible early re-stenosis [21]. In our study, only 6% of treated patients had near occlusions. There were no peri-interventional complications in this group of patients. The ESVS and German-S3 guidelines tend to stand against CAS for this group patients, but do not give a clear recommendation [3,5].

The lack of experience of the interventionalist or centers in randomized multi-center trials has always been a topic of discussion, and it has been postulated that this was a possible reason for the poor CAS outcomes in this trial [3]. Great variability can also be observed in terms of the requirements for carrying out the intervention. In some trials, the interventionalists needed almost no experience and in others a lot of experience. For example, in ICSS, a minimum number of ≥50 stent implantations but only ≥10 CAS had to be fulfilled in order to be able to participate in that study [29]. In EVA-3S, the minimum number to participate in the study was 12 CAS or 35 supraaortic interventions [8], for SPACE, ≥25 PTAs but not necessarily CAS interventions [30]. On the other hand, CREST was the only study in which not only intervention numbers were required, but also participating surgeons and interventionalists had to meet defined training and experience requirements to participate in the study [31].

Currently, there is plenty of information supporting the theory that experience of interventionalists or centers is crucial to perform CAS in a safe manner. At the same time there are completely different cut-off values by which “experience” is considered. A CSTC meta-analysis of three large European RCTs (EVA-3S, SPACE, and ICSS) revealed that the 30-day risk of stroke or death did not differ according to operator lifetime CAS experience. In contrast, the 30-day risk of stroke or death was significantly higher in patients treated by operators with yearly low volume (3.2 CAS/year) compared to patients treated by high annual volume operators (>5.6 CAS/year) [29]. A Health Insurance Database study from Taiwan with >3500 CAS showed that CAS being performed in high-volume hospitals (>20 CAS procedures/year) was associated with lower 30-day stroke rates compared to those in low-volume hospitals [32]. In a single-center study with >2000 CAS interventions, it was demonstrated that surgeons with >100 CAS interventions had fewer perioperative strokes compared to those with fewer interventions. On the other hand, surgeons with <50 CAS procedures were a risk factor for increased rate of stroke [33]. The requirement regarding experience plays a very important role in update trials like CREST-2, SPACE-2, ACST-2. Unfortunately, with the available data it is still not possible to provide a minimum number of procedures by which CAS could be safely performed. In Germany, the guidelines recommend that CAS interventions should be performed by qualified and experienced physicians. In addition, CAS should be performed in centers with at least 10 procedures/year [3]. In our center, approximately 30 elective CAS procedures/year were done. The supra-aortic interventions are exclusively done by highly experienced endovascular specialists. Two fifths of surgeons had already performed more than 100 CAS procedures before the start of the study and were present and active during the entire research period. The three other surgeons had over 10 years of experience with central and peripheral vascular interventions. They joined the CAS-team within the nine years of the study, while one of them dropped out before the end of this period. Each had performed at least >1000 interventions at baseline. The first 20 CAS interventions were supervised by one of the two more experienced operators, ensuring that the safety and quality level was maintained. Our CAS procedures were distributed annually among a maximum of three to four interventionalists. In our institution, of the three neurological events, one occurred in 2015 and two in 2020. All three neurological events occurred in cases performed by the most experienced surgeons. Thus, our results do not correspond with those from Setacci et al.

We believe that due to the randomization and the very broad inclusion criteria of the large clinical trials, presumably this patient would not have been stented in experienced centers under the above standard conditions. This basic problem also persists in the update trials.

Another problem is the variety of available devices and interventional steps performed. In some randomized trials, there were specifications as to which systems (stent type and EPS) had to be used, with which some groups had limited experience. In other studies, there was a free choice of stent and EPS, which of course made comparison difficult because the stent designs differ fundamentally. Some authors describe better results with closed-cell design stents rather than with open-cell design [34,35,36]. Other studies found no difference [37,38]. Currently, data are still lacking on which to make a concrete recommendation. In the German S3-guideline, only self-expanding stents are recommended, but no recommendation is made regarding the design [3]. On the other hand, the ESVS guideline gives a weak recommendation towards using the new technology for dual-layer stents. Whether the stent design has an impact on outcomes remains unclear.

Regarding the use of EPS, there is no unanimous recommendation in the existing literature. Meta-analysis and data from EVA-3S, CREST, and ACT-I trials showed a significant reduction in stroke rates given the use of protective systems [6,8,16,39]. EPS have been used in the CREST trial in as much as 96% of the CAS-treated group. This is one of the reasons why it is believed that such a low rate of strokes was observed. CREST without EPS showed an approximately four-fold higher risk of neurological events and EVA-3S an approximately three-fold higher risk [6,8]. In a subgroup analysis of the SPACE-1- trial, it was not possible to demonstrate the benefits in symptomatic patients in whom EPS for CAS have been used compared to CAS without protection [36]. All these studies used a non-occluding filter system placed distally to the stenosis. However, it must also be mentioned that no RCTs have yet been carried out to compare CAS with EPS against CAS without, nor to compare EPS placed distally against those placed proximally (reversed flow systems) to the stenosis. The Society for Vascular Surgery VQI TCAR (transcarotid artery revascularization) Surveillance Project recently showed promising perioperative results for TCAR compared to CAS [40]. The S3-German and ESVS guideline does not give any recommendation in this regard, but tends to use an EPS.

Studies in which inhomogeneous materials were used were also particularly criticized. In the CAVATAS trial, some of the patients of the interventional arm were treated with percutaneous transluminal angioplasty (PTA) alone, whereas others were treated using stent-assisted PTA. Here, the interventionalists were allowed to choose the stents [41]. In ICSS and EVA-3S, a mixture of different stents (closed cell and open cell) and filter systems were used. In EVA-3S, an embolic protection system was used in 92% of cases, and in ICSS, 72% of cases [8,9]. In the SPACE trial, significantly fewer EPS were used. This was only used in 27% of CAS [7]. In the CREST trial, on the other hand, the use of the materials was homogeneous through the use of the RX AccuLink Stent and RX AccuNet EPS (Abbott Vascular) [6]. In this study, the same materials such as wires, catheters, sheath, filter system, balloon, stents, and closure system were used for all carotid interventions. Thus, great experience with these systems has been developed over the years. In respect to this, our results support the work of Fiehler et al. who demonstrated that experience with the armamentarium often has a greater influence on outcome than the method itself [42].

Most trials have focused almost exclusively on symptomatic carotid stenosis. The SAPPHIRE, CREST, and ACT-I trials have also included asymptomatic patients, so the evidence in favor of stenting in asymptomatic stenosis is still poor [6,13,16]. However, the reality is different. For example, in Germany, more than 55% of CAS are done in asymptomatic patients [43]. The current studies tend to focus mainly on the asymptomatic patient group such as SPACE-2, CREST 2, ACST-2, ECST-2 and ACTRIS.

The present study has limitations. From our point of view, possible weaknesses are the retrospective design of the study, the very short follow up period, and that only periprocedural complications were recorded. Other limitations of the study include the conclusions being drawn on the basis of a small number of cases and the lack of comparison of CAS with patients undergoing CEA.

In conclusion, we support the statement that CAS is a low-risk alternative to CEA. In comparison to the existing randomized trials, we were able to show that through careful patient selection based on the anatomical criteria, the standardization of the procedure by using a specific treatment protocol and the execution of CAS by highly experienced and qualified surgeons could end up giving a very low complication rate. Patient- and age-related anatomical features can represent an additional technical challenge at the time of performing CAS, but the key lies in recognizing which patients are at greater risk for CAS and which are not.

## Figures and Tables

**Table 1:** Characteristics of the study population.

Patient Characteristics	Total
**CAS procedures**	202
**Symptoms**	
asymptomatic	156 (77%)
symptomatic	46 (23%)
**Age (years) mean ± SD**	66 ± 9
**Gender**	
Male	166 (82%)
Female	36 (18%)
**Risk Factors**	
Hypertension	176 (87%)
Dyslipidemia	122 (60%)
Diabetes mellitus	64 (32%)
Coronary artery disease	92(46%)
Peripheral arterial disease	46 (22%)
Atrial fibrillation	36 (18%)
Obesity	56 (28%)
Current smoker	65 (32%)
**Preoperative medication**	
ASS	190 (94%)
Statins	128 (63%)
Clopidogrel loading dose 300 mg	202 (100%)

**Table 2:** Lesion characteristics.

Lesion Characteristics	Total
**Side**	
Right	123 (61%)
Left	79 (39%)
**Degree of stenosis** (MW ± SD)	
Ipsilateral	81% ± 11
Contralateral	25% ± 36
**Stenosis length** (MW ± SD)	
Mean	25 ± 8 mm
**Vessel Diameter** (MW ± SD)	
CCA	7 ± 1 mm
ICA	5 ± 0.7 mm
**Aortic arch type**	
Type I	81 (40%)
Type II	121 (60%)
**Angle AA—supra-aortic vessel** (MW ± SD)	
Type I AA—Brachiocephalic trunk	77° ± 13°
Type I AA—left CCA	68° ± 15°
Type II AA—Brachiocephalic trunk	70° ± 16°
Type II AA—left CCA	60° ± 12°
**Angle CCA-ICA** (MW ± SD)	24° ± 8°
**Plaque classification**	
Lipid	54 (39%)
Fibrous	61 (44%)
Calcified	23 (17%)
**Vessel course variations**	
Elongation	40 (20%)
Kinking	0
Coiling	0
**Type of Lesion**	
Eccentric	174 (86%)
Concentric	15 (7%)
Carotid near occlusion	13 (6%)

AA: aortic arch; CCA: common carotid artery; ICA: internal carotid artery.

**Table 3:** Stand of this study.

	CAS	CEA
**Randomized Multicenter Trials**		
CAVATAS	6.4%	5.9%
SAPPHIRE	4.4%	9.9%
EVA-3S	9.6%	3.9%
SPACE	6.8%	6.3%
ICSS	8.5%	5.2%
CREST	5.2%	4.5%
ACT-I	2.9%	1.7%
ACST-II	3.9%	3.2%
**Single Center Trials without Randomization**		
Werner, M. et al. [10]	2.0%	-
Setacci, C. et al. [11]	1.5%	2.1%
Mayoral Campos et al. [12]	2.3%	-
Bonifatius Hospital Lingen *	1.5%	-

Refs. [6,7,8,9,10,11,12,14,15,16] * At Bonifatius Hospital Lingen the results are only perioperative.

## Data Availability

Not applicable.
